# Effects of gestational diabetes mellitus and assisted reproductive technology treatment on the risk of preterm singleton birth

**DOI:** 10.3389/fnut.2022.977195

**Published:** 2022-09-14

**Authors:** Rui Gao, Ke Zhao, Jiaxin Zhou, Xiaona Wang, Ting Liu, Shaoyan Lian, Jieying Li, Yuanyan Huang, Chuhui Qiu, Yuxiao Wu, Jiang He, Chaoqun Liu

**Affiliations:** ^1^Shenzhen Cadre and Talent Health Institute (Shenzhen Talent Institute), Shenzhen, Guangdong, China; ^2^Shenzhen Birth Cohort Study Center, Department of Science and Education, Nanshan Maternity and Child Healthcare Hospital of Shenzhen, Shenzhen, Guangdong, China; ^3^Department of Nutrition, School of Medicine, Jinan University, Guangzhou, Guangdong, China; ^4^Department of Epidemiology, College of Public Health, University of Iowa, Iowa City, IA, United States; ^5^Department of Mathematics and Physics, School of Biomedical Engineering, Southern Medical University, Guangzhou, Guangdong, China

**Keywords:** gestational diabetes mellitus, assisted reproductive technology, preterm birth, singleton birth, NVSS

## Abstract

**Background:**

Although assisted reproductive technology (ART) plays a critical role in reducing infertility, ART pregnant women are reported at higher risk of preterm birth (PTB). Besides, women undergoing ART encounter a higher risk of developing gestational diabetes mellitus (GDM). However, existing studies on the combined effect of ART treatment and GDM on PTB risk are sparse.

**Methods:**

This population-based retrospective cohort study used nationwide birth certificate data from the US National Vital Statistics System 2015-2019. All mothers who had a singleton live birth without pre-pregnancy diabetes were included. Multivariable logistic regression models were used to estimate the odds ratio (OR) of PTB.

**Results:**

We finally included 18,140,241 American mother-infant pairs. The overall rate of PTB was 7.92% (n = 1,436,328). The PTB rate for non-ART mothers without GDM, ART mothers without GDM, non-ART mothers with GDM, and ART mothers with GDM were 7.67, 10.90, 11.23, and 14.81%, respectively. The incidence of GDM in ART mothers (10.48%) was significantly higher than in non-ART mothers (6.26%). After adjusting for potential confounders, compared with non-ART mothers without GDM, the PTB risk was significantly increased for ART mothers without GDM (AOR: 1.47, 95% CI 1.44-1.50), non-ART mothers with GDM (AOR:1.35, 95% CI 1.34-1.36) and ART mothers with GDM (AOR: 1.82, 95% CI 1.74-1.90) respectively, showing an increasing tendency. This phenomenon was stable among mothers in all groups of mothers older than 25 years.

**Conclusion:**

To prevent PTB, effective approaches for the prevention of GDM are crucial to mothers who conceived through ART.

## Introduction

Preterm birth (PTB) is defined as delivery before 37 weeks of gestation. According to WHO, approximately 15 million infants (11%) are born preterm annually worldwide, of which more than 1 million children die before the age of 5 years due to PTB and its complications (1). Unfortunately, to date, few interventions are efficient to reduce PTB. Assisted reproductive technology (ART) is a relatively mature procedure to address infertility, its demand is increasing worldwide (2). To date, more than 8 million babies have been born after ART globally (3). According to the Centers for Disease Control and Prevention (CDC), in 2019, 2.1% of all infants born in the United States were conceived through ART (4). The PTB rate was higher among infants conceived with ART (24.4%) than among all infants born in the total birth population (10.2%). Aside from the direct role of multifetal pregnancies, even among ART singletons, the rate of PTB was higher among ART-conceived infants (15.4%) than among all infants (8.5%). Similar results were found in a meta-analysis of 50 cohort studies and indicated that singleton pregnancies created with ART experienced a significantly increased risk of PTB (RR 1.71, 95% CI 1.59–1.83) (4). Although ART may help infertile couples achieve pregnancy, the attendant risk of PTB can bring an extra burden to the family. However, the reasons for the greater increase in PTB among ART pregnancies remain obscure. This ART-PTB association may be fully or partially confounded by maternal health during pregnancy.

Gestational diabetes mellitus (GDM) is the most common pregnancy complication defined as glucose intolerance first identified during pregnancy (5), that is affecting 7–25% of pregnancies worldwide (6). It is well recognized now that GDM is a critical risk factor for spontaneous and indicated PTB (< 37 weeks of gestation) (7–9). Despite advances in the care of women with GDM, the odds of PTB were 30% higher than in those without diabetes (10). Maternal hyperglycemic states during pregnancy with alterations in insulin and glucose metabolism can result in an adverse intrauterine environment for the developing fetus (11). Moreover, some early delivery in GDM women may be recommended because the baby is large. Several research reported that pregnant women with a history of fertility problems, particularly from ovulation disorders and tubal blockage, are at increased risk of GDM, reaching an approximate prevalence of 11-40% in women undergoing ART (12–15). A study by Szymanska et al. suggested that IVF patients may develop GDM earlier in pregnancy because of higher first trimester fasting glucose levels (16). Although previous studies have suggested an association of PTB with GDM and ART separately, rarely studies have investigated the PTB risk in ART women combined with GDM.

Considering the increasing usage of ART and growing awareness of ART pregnant women at high risk of developing GDM, we aim to use a large population-based study to explore the combined effect of ART treatment and GDM on the risk of preterm singleton birth.

## Materials and methods

### Study design and data sources

The data were from the US National Vital Statistics System (NVSS), an extensive data archive accessible to the public, which was conducted by the National Center for Health Statistics (NCHS) at the Centers for Disease Control and Prevention (CDC). NVSS collects and publishes nationwide data on births in all the 50 US states and the District of Columbia from birth certificates according to Federal law. In this population-based study, we used birth data from Jan 1, 2015, to Dec 31, 2019 (NVSS 2015-2019), including all mothers (*n* = 18,140,241) who had a live singleton birth without prepregnancy diabetes, excluding women with incomplete data on ART treatment, GDM and gestational age at birth. The screening process is shown in Supplementary Figure 1. This study was approved as exempt from review by the Institutional Review Board at Jinan University due to the use of de-identified data from the open database. The conduct and reporting of this study followed the reporting guidelines in the Strengthening the Reporting of Observational Studies in Epidemiology statement.

### Exposure measurement and outcome ascertainment

Information on ART treatment (*in vitro* fertilization (IVF), gamete intrafallopian transfer (GIFT), zygote intra-fallopian transfer (ZIFT)), GDM, and gestational age were collected directly from the medical record by the facility worksheet. According to WHO, PTB was further subdivided into extremely preterm (< 28 weeks), very preterm (28-31 weeks), and moderate preterm (32-36 weeks) (17).

### Covariates measures

Information on maternal age (<25, 25-29, 30-34, 35-39, > 40 years), maternal race/ethnicity (Hispanic, non-Hispanic black, non-Hispanic whites, and others), maternal education (lower than high school, high school, higher than high school, and unknown), marital status (married, unmarried, and unknown), pre-pregnancy body mass index (underweight < 18.5 kg/m^2^, normal weight 18.5-24.9 kg/m^2^, overweight 25.0-29.9 kg/m^2^, obesity ≥ 30.0 kg/m^2^), Smoking before or during pregnancy (yes, no) were collected using the mother’s worksheet. Information on parity (1, 2, 3, ≥ 4), pre-pregnancy hypertension (yes, no), previous preterm birth (yes, no, nulliparous), initiation of prenatal care (no prenatal care, 1st–3rd month, 4th–6th month, 7th–final month, or missing), gestational hypertension or preeclampsia or eclampsia (yes, no) and infant sex (male or female) were collected using the facility worksheet.

### Statistical analysis

Baseline characteristics are presented as numbers and proportions. Comparisons between categorical variables were tested using chi-square tests. This retrospective cohort is divided into four groups according to ART and GDM: non-ART mothers without GDM, ART mothers without GDM, non-ART mothers with GDM, and ART mothers with GDM. Logistic regression models were used to estimate odds ratios (ORs) and 95% confidence intervals (95% CIs) for PTB, after adjusting for potential confounders, including maternal age, race/ethnicity, education, marital status, parity, pre-pregnancy body mass index, hypertension before pregnancy, previous history of PTB, smoking before pregnancy, smoking during pregnancy, initiation of prenatal care, gestational hypertension or preeclampsia & eclampsia and infant sex. We further conducted secondary analyses stratified by maternal age (< 25, 25-34, ≥ 35 years old). *P* for interaction was calculated on the basis of multivariable logistic regression models with multiplicative interaction term (age/pre-pregnancy body mass index *exposure groups). All analyses were performed with R statistical software (version 3.6.4) and GraphPad Prism 8 (GraphPad Software, San Diego, CA). A two-sided *P* < 0.05 was considered statistically significant.

## Results

### Baseline characteristics

Table 1 details the baseline characteristics of the study population, according to ART and GDM categories. Among all the participants, ART mothers account for 0.80% (*n* = 145,557), GDM mothers account for 6.21% (*n* = 1,125,651). ART mothers were more likely to be elder, non-Hispanic white, higher educated, married primiparous, and earlier initiation of prenatal care and had gestational hypertension or preeclampsia or eclampsia, while less smoking before or during pregnancy than non-ART mothers without GDM. Mothers with GDM were more likely to be elder, non-Hispanic white, obesity and hypertension before pregnancy, and earlier initiation of prenatal care than non-ART mothers without GDM. We also found the incidence of GDM in ART mothers (10.48%) was higher than in non-ART mothers (6.17%) (Supplementary Table 1). The PTB rate for non-ART mothers without GDM, ART mothers without GDM, non-ART mothers with GDM, and ART mothers with GDM were 7.67, 10.90, 11.23, and 14.81%, respectively, showing an increasing tendency (Table 1). Supplementary Table 2 shows the characteristics of the study population according to PTB. Comparison between any two or more groups was performed by the Chi-square test (Supplementary Table 3). We found that ART mothers with GDM had the highest rate of PTB compared to the other three groups.

**TABLE 1:** Characteristics of the study population, according to received assisted reproductive technology and/or developed gestational diabetes mellitus.

Variables	*N*	GDM or ART			
		non-ART without GDM	ART only	GDM only	ART with GDM
		
Overall	18140241	16884295	130295	1110389	15262
**Age, years, *n* (%)**					
< 25	4622407 (25.48)	4476146 (26.51)	1183 (0.91)	144992 (13.06)	86 (0.56)
25-29	5283616 (29.13)	4991899 (29.57)	12218 (9.38)	278553 (25.09)	946 (6.20)
30-34	5111379 (28.18)	4696053 (27.81)	42751 (32.81)	368427 (33.18)	4148 (27.18)
35-39	2556189 (14.09)	2256582 (13.36)	45756 (35.12)	248093 (22.34)	5758 (37.73)
≥ 40	566650 (3.12)	463615 (2.75)	28387 (21.79)	70324 (6.33)	4324 (28.33)
**Race or ethnicity, *n* (%)**					
Hispanic	4326096 (23.85)	4023171 (23.83)	10386 (7.97)	291139 (26.22)	1400 (9.17)
Non-Hispanic Black	2590208 (14.28)	2455445 (14.54)	6113 (4.69)	127901 (11.52)	749 (4.91)
Non-Hispanic White	9356729 (51.58)	8743653 (51.79)	92120 (70.70)	512137 (46.12)	8819 (57.78)
Other	1867208 (10.29)	1662026 (9.84)	21676 (16.64)	179212 (16.14)	4294 (28.14)
**Education levels, *n* (%)**					
Lower than high school	2412989 (13.3)	2258681 (13.38)	1179 (0.90)	152871 (13.77)	258 (1.69)
High school	4599029 (25.35)	4326654 (25.63)	6827 (5.24)	264514 (23.82)	1034 (6.77)
Higher than high school	10897734 (60.07)	10087670 (59.75)	118347 (90.83)	678140 (61.07)	13577 (88.96)
Missing	230489 (1.27)	211290 (1.25)	3942 (3.03)	14864 (1.34)	393 (2.58)
**Marital status, *n* (%)**					
Yes	10031521 (55.3)	9240066 (54.73)	108754 (83.47)	670267 (60.36)	12434 (81.47)
No	6788854 (37.42)	6425768 (38.06)	8939 (6.86)	353031 (31.79)	1116 (7.31)
Missing	1319866 (7.28)	1218461 (7.22)	12602 (9.67)	87091 (7.84)	1712 (11.22)
**Parity, *n* (%)**					
1	6979846 (38.48)	6521796 (38.63)	78770 (60.46)	369858 (33.31)	9422 (61.74)
2	5783078 (31.88)	5394947 (31.95)	35213 (27.03)	349002 (31.43)	3916 (25.66)
3	3070294 (16.93)	2849688 (16.88)	9877 (7.58)	209592 (18.88)	1137 (7.45)
≥ 4	2251757 (12.41)	2064878 (12.23)	6214 (4.77)	179900 (16.20)	765 (5.01)
Missing	55266 (0.3)	52986 (0.31)	221 (0.17)	2037 (0.18)	22 (0.14)
**Pre-pregnancy body mass index, *n* (%)**					
Underweight	596142 (3.29)	575725 (3.41)	3166 (2.43)	17007 (1.53)	244 (1.60)
Normal weight	7654583 (42.2)	7306013 (43.27)	67457 (51.77)	275955 (24.85)	5158 (33.80)
Overweight	4656694 (25.67)	4333373 (25.67)	32847 (25.21)	286189 (25.77)	4285 (28.08)
Obesity	4770149 (26.3)	4234008 (25.08)	24553 (18.84)	506244 (45.59)	5344 (35.02)
Missing	462673 (2.55)	435176 (2.58)	2272 (1.74)	24994 (2.25)	231 (1.51)
**Pre-pregnancy hypertension, *n* (%)**					
Yes	337634 (1.86)	283421 (1.68)	3640 (2.79)	49670 (4.47)	903 (5.92)
No	17802607 (98.14)	16600874 (98.32)	126655 (97.21)	1060719 (95.53)	14359 (94.08)
**Previous preterm birth, *n* (%)**					
Yes	580725 (3.2)	522311 (3.09)	3984 (3.06)	53793 (4.84)	637 (4.17)
No	10579751 (58.32)	9840254 (58.28)	47543 (36.49)	686751 (61.85)	5203 (34.09)
Nulliparous	6979765 (38.48)	6521730 (38.63)	78768 (60.45)	369845 (33.31)	9422 (61.74)
**Smoking before pregnancy, *n* (%)**					
Yes	1612037 (8.89)	1513497 (8.96)	1345 (1.03)	96970 (8.73)	225 (1.47)
No	16427864 (90.56)	15275982 (90.47)	128612 (98.71)	1008285 (90.80)	14985 (98.19)
Missing	100340 (0.55)	94816 (0.56)	338 (0.26)	5134 (0.46)	52 (0.34)
**Smoking during pregnancy, *n* (%)**					
Yes	604131 (3.33)	570083 (3.38)	273 (0.21)	33718 (3.04)	57 (0.37)
No	16783121 (92.52)	15604986 (92.42)	129063 (99.05)	1033977 (93.12)	15095 (98.91)
Missing	752989 (4.15)	709226 (4.20)	959 (0.74)	42694 (3.84)	110 (0.72)
**Initiation of prenatal care, *n* (%)**					
No prenatal care	295147 (1.63)	288016 (1.71)	299 (0.23)	6804 (0.61)	28 (0.18)
1th to 3th month	13628169 (75.13)	12632570 (74.82)	114845 (88.14)	867389 (78.12)	13365 (87.57)
4th to 6th month	2932944 (16.17)	2750660 (16.29)	11059 (8.49)	169851 (15.30)	1374 (9.00)
7th to final month	814195 (4.49)	767700 (4.55)	1834 (1.41)	44421 (4.00)	240 (1.57)
Missing	469786 (2.59)	445349 (2.64)	2258 (1.73)	21924 (1.97)	255 (1.67)
**Gestational hypertension or preeclampsia & eclampsia, *n* (%)**					
Yes	1040072 (6.16)	13172 (10.11)	137208 (12.36)	2300 (15.07)	1040072 (6.16)
No	15844223 (93.84)	117123 (89.89)	973181 (87.64)	12962 (84.93)	15844223 (93.84)
**Infant sex, *n* (%)**					
Male	9281667 (51.17)	8632431 (51.13)	66522 (51.05)	574891 (51.77)	7823 (51.26)
Female	8858574 (48.83)	8251864 (48.87)	63773 (48.95)	535498 (48.23)	7439 (48.74)
**preterm birth, *n* (%)**					
Yes	1436328 (7.92)	1295163 (7.67)	14200 (10.90)	124704 (11.23)	2261 (14.81)
No	16703913 (92.08)	15589132 (92.33)	116095 (89.10)	985685 (88.77)	13001 (85.19)

ART, assisted reproductive technology; GDM, gestational diabetes mellitus. The percentage in bracket was the composition ratio of each horizontal item. Chi-square test showed that the inter-group P value of each variable was less than 0.05.

### Effects of assisted reproductive technology and gestational diabetes mellitus on preterm birth

We used a multivariable logistic regression model to assess the effects of ART and GDM on PTB (Table 2). After adjusting for confounding factors, compared with non-ART mothers without GDM, the overall PTB risk was significantly increased for ART mothers without GDM, non-ART mothers with GDM, and ART mothers with GDM respectively, and ART mothers with GDM had the highest PTB risk. Furthermore, subgroup analysis by gestational age (extremely preterm <28 weeks, very preterm 28-31 weeks, and moderate preterm 32-36 weeks) was also performed. We also found that ART mothers with GDM had the highest risk ratios for moderate and very PTB. The OR values kept increasing as the severity of PTB increased in ART mothers without GDM. However, having GDM only was mainly associated with an increased the risk of moderately PTB, unexpectedly a lower risk of being severely preterm.

**TABLE 2:** Effects of assisted reproductive technology and/or gestational diabetes mellitus on preterm birth.

	Preterm birth, OR (95% CI)	Preterm birth categories		
		Moderately preterm birth, OR (95% CI)	Very preterm birth, OR (95% CI)	Extremely preterm birth, OR (95% CI)
ART only	1.47 (1.44-1.50)	1.43 (1.40-1.46)	1.56 (1.47-1.65)	1.96 (1.83-2.11)
GDM only	1.35 (1.34-1.36)	1.43 (1.42-1.44)	1.01 (0.99-1.03)	0.56 (0.54-0.59)
ART with GDM	1.82 (1.74-1.90)	1.83 (1.74-1.93)	2.05 (1.80-2.33)	1.21 (0.95-1.55)

ART, assisted reproductive technology; GDM, gestational diabetes mellitus; OR, odds ratio; CI, confidence interval.

All models are adjusted for age, race/ethnicity, education, marital status, parity, pre-pregnancy body mass index, hypertension before pregnancy, previous history of preterm birth, smoking before pregnancy, smoking during pregnancy, initiation of prenatal care, gestational hypertension or preeclampsia & eclampsia and infant sex.

### Subgroup analysis by maternal age and prepregnancy body mass index

Considering maternal age as an important confounding factor, we conducted secondary analyses stratified by maternal age (< 25, 25-34, ≥ 35 years old) (Table 3). We found that ART mothers with GDM had the significantly highest rate of PTB in all groups of mothers older than 25 years. Scarified analysis by prepregnancy BMI (underweight < 18.5 kg/m^2^, normal weight 18.5-24.9 kg/m^2^, overweight 25.0-29.9 kg/m^2^, obesity ≥ 30.0 kg/m^2^) also showed the same tendency that ART mothers with GDM had the highest rate of PTB in those with prepregnancy normal weigh, overweight and obesity groups (Table 4).

**TABLE 3:** Effects of assisted reproductive technology and/or gestational diabetes on preterm birth according to age.

Age	Groups	Case/No.	Adjusted OR (95% CI)	*P* for interaction	*P* for chi-square *test*
< 25	ART only	140/1183	1.73 (1.45-2.08)	< 0.0001	
	GDM only	16124/144992	1.40 (1.37-1.42)		< 0.0001
	ART with GDM	14/86	2.38 (1.33-4.29)		
25-34	ART only	5672/54969	1.63 (1.58-1.67)		
	GDM only	68593/646980	1.38 (1.37-1.39)		< 0.0001
	ART with GDM	717/5094	2.09 (1.93-2.27)		
≥ 35	ART only	8388/74143	1.39 (1.35-1.42)		
	GDM only	39987/318417	1.25 (1.24-1.27)		< 0.0001
	ART with GDM	1530/10082	1.66 (1.57-1.76)		

ART, assisted reproductive technology; GDM, gestational diabetes mellitus, OR, odds ratio, CI, confidence interval.

Race/ethnicity, education, marital status, parity, pre-pregnancy body mass index, hypertension before pregnancy, previous history of preterm birth, smoking before pregnancy, smoking during pregnancy, initiation of prenatal care, gestational hypertension or preeclampsia & eclampsia and infant sex were adjusted in this model.

**TABLE 4:** Effects of assisted reproductive technology and/or gestational diabetes on preterm birth according to pre-pregnancy body mass index.

Pre-pregnancy BMI	Groups	Case/No.	Adjusted OR (95% CI)	*P* for interaction	*P* for chi-square test
Underweight	ART only	305/3166	1.44 (1.28-1.62)	< 0.0001	
	GDM only	1787/17007	1.22 (1.16-1.29)		0.0002
	ART with GDM	30/244	1.94 (1.31-2.87)		
Normal weight	ART only	6188/67457	1.61 (1.57-1.66)		
	GDM only	25449/275955	1.37 (1.35-1.38)		< 0.0001
	ART with GDM	630/5158	2.06 (1.89-2.24)		
Overweight	ART only	3750/32847	1.71 (1.65-1.77)		
	GDM only	29671/286189	1.39 (1.37-1.41)		< 0.0001
	ART with GDM	636/4285	2.14 (1.96-2.33)		
Obesity	ART only	3573/24553	1.64 (1.58-1.71)		
	GDM only	64026/506244	1.39 (1.38-1.41)		< 0.0001
	ART with GDM	918/5344	1.90 (1.76-2.04)		

ART, assisted reproductive technology; GDM, gestational diabetes mellitus; BMI, body mass index; OR, odds ratio; CI, confidence interval.

Race/ethnicity, education, marital status, parity, pre-pregnancy body mass index, hypertension before pregnancy, previous history of preterm birth, smoking before pregnancy, smoking during pregnancy, initiation of prenatal care, gestational hypertension or preeclampsia & eclampsia and infant sex were adjusted in this model.

## Discussion

In the present study based on the 2015-2019 NVSS singleton livebirths databases, we found that either GDM or ART use was associated with an increased risk of PTB, especially ART mothers with GDM were at maximum risk of PTB. Moreover, ART mothers had a higher incidence of GDM than non-ART mothers. Subgroup analysis further found that ART-GDM mothers had the highest risk of mid-to-late PTB, indicating the timing of PTB at most risk. Moreover, ART-GDM mothers had the highest rate of PTB in all groups of mothers older than 25 years. Therefore, ART pregnancies need to pay special attention to GDM for preventing PTB.

In this study, we found that after adjustment for potential confounding factors, ART mothers with GDM had the highest risk of PTB compared to the other three groups. This is an important finding, as most of the previous studies report separate estimates of the causal effect of ART and GDM on PTB, very few studies have investigated the PTB risk in ART women combined with GDM. Although it is clear that both ART and GDM are independent risk factors for PTB (7–9, 18), little is known about the risk of PTB among ART-GDM pregnancies. A similar result was found in the previous study (Kouhkan et al. (19)), compared to spontaneously conceived pregnancies, ART-GDM pregnancies are delivered 2 weeks earlier and those conceived by ART and SC-GDM are delivered 1 week earlier (20). But obvious limitations of their study include a single-center study in Tehran and with small sample size, consisting of only 260 ART and 314 SC, 135 and 152 women were GDM women, respectively. The GDM rates in this cohort are significantly higher than the general population, so the biases may affect the representativeness of their findings. Consequently, we consider our results representative of a more accurate estimate, and PTB prevention efforts should have an increased focus on ART-GDM pregnancies.

Evidence continues to accumulate that women undergoing ART often encounter major risk factors for GDM, such as advanced maternal age, obesity, multiple pregnancies, and polycystic ovary syndrome (PCOS), suggesting a link between GDM and ART (13–15). A recent meta-analysis by Mohammadi et al. including 48 studies with 91,487 pregnancies conceived through ART and 2,525,234 spontaneously conceived indicates that the use of ART treatment is associated with a 1.51-fold increase in GDM (21). This was also confirmed in our study that ART women were at higher risk of GDM, reflected in 10.49% GDM rate in ART mothers with singleton pregnancies and 6.17% GDM rate in non-ART group. We further tried to understand the reasons behind this observation. Similarly, compared to the non-ART mothers, ART mothers were observed for older age, and higher prepregnancy BMI, which are known to promote insulin resistance, and insulin resistance increases the risk for developing GDM. Another pivotal factor that must be mentioned is PCOS, the most common cause of female infertility, and 62% of the women with persistent PCOS underwent ART treatment. Previous researchers revealed that women with PCOS had a more than twofold increased odds of GDM compared with women without PCOS (22, 23). Even though studies that specifically excluded PCOS women still reported ART mothers had a higher risk of GDM than non-ART mothers (15). Kouhkan et al. performed a nested case-control study including 270 ART women with singleton pregnancies (consisting of 135 GDM and 135 non-GDM women) also showed that the route of progesterone administration (OR 2.28, 95% CI 1.27–4.09), previous ovarian hyper-stimulation syndrome (OHSS) risk (OR 2.40, 95% CI 1.34–4.31) and history of PCOS (OR 2.76, 95% CI 1.26–6.06) seem to be putative risk factors for GDM in women whose pregnancies conceived by ART (19). Given the complexity of ART, multiple factors affect PTB risk. Besides, many of these risk factors for ART and GDM are also risk factors for PTB. Together, these findings suggest that ART population is at higher risk for developing of GDM, ART and GDM might exert a combined effect on the risk of PTB.

Moreover, we stratified the outcomes into subcategories of PTB: moderately preterm (32–36 weeks), very preterm (28-31 weeks), and extremely preterm (<28 weeks). Notably, ART-GDM mothers had the highest risk of PTB from 28 to less than 37 weeks (mid-to-late gestation) compared to the other three groups after adjustment for potential confounding factors. In more detail, compared to non-ART mothers without GDM, ART-only mothers had a high risk of PTB in early, middle, and late pregnancy, and the ratio increased with increasing severity of PTB. Furthermore, using data from the Quebec Pregnancy Cohort (QPC) included 57,624 pregnancies, recent results from Gorgui et al. (24) report similar findings. They also observed a trend across PTB categories that the use of ovarian stimulators or ART or both were associated with an increased risk of late (AOR:1.61, 95%CI 1.03–2.51), moderate (AOR:1.59, 95%CI 0.94–2.68) and extremely (AOR: 2.39, 95%CI 1.30–4.39) PTB (24). Since the inclusion of ovarian stimulators as a variable, the PTB risk was slightly higher than those observed in our study. Nonetheless, we discovered that GDM-only mothers were more likely to have a moderate PTB. Disentangling this question is not an easy feat. The majority of recent research has found that GDM is linked to rapid fetal growth (25), putting big newborns at risk of premature birth. Maternal insulin resistance, in addition to the consequences of maternal hyperglycemia, causes excessive fetal growth by increasing the placental transfer of additional growth substrates such as amino acids and lipids (26). Chronic fetal hyperinsulinism causes increased fetal substrate absorption, which raises tissue oxygen demand. This causes relative fetal hypoxia, which raises the risk of iatrogenic PTB or fetal mortality within the womb (27). In addition, GDM mothers are more likely to have pre-eclampsia, premature placental aging, placental abruption, and other complications in late pregnancy, resulting in late-PTB. Surprisingly, having GDM only was associated with a lower risk of being severely preterm. However, the evidence for the association between GDM and early PTB is inconsistent and often hampered by small sample size. A study from Medical Center’s (SUMC) birthing center yielded similar results as ours, which comprised 334,415 deliveries between 1991 and 2018 and found that diabetes mellitus had an inverse connection with the risk of early PTB (28). But Geurtsen et al. indicated that maternal early-pregnancy non-fasting glucose levels were not associated with PTB or delivery complications (29). GDM is generally diagnosed at 24-28 weeks of gestation, and the onset of GDM typically occurs in the second trimester of pregnancy, when progesterone levels are high (30). Besides, fetal insulin is a key fetal growth factor, which is inactive until the onset of corticoid action in the second trimester that why glycogen storage does not occur before the 27th week (31). In addition, threatened miscarriage and stillbirth are frequent during the first trimester, but not being considered together with extremely PTB and may introduce some bias. To date, the mechanisms underlying the association of GDM on fetal during the first trimester is unclear and require further study. Furthermore, since maternal age is an important factor affecting PTB, we found that ART-GDM mothers had the highest rate of PTB in over 25 age groups, suggesting no effect of age on the primary outcome. Overall, women whose pregnancies conceived through ART should be monitored for GDM, which directly and indirectly increases the risk of mid-to-late PTB.

There are several strengths in this study. Firstly, this study provides the first nationally representative estimates of PTB risk in ART-GDM pregnancies and can be considered more accurate. In addition, the availability of many clinical variables and large sample size allowed us to perform a detailed adjusted analysis that accounts for many important potential confounding variables such as prepregnancy BMI and race, some of which were not accounted for in previous studies (24, 28).

We acknowledge that there are several limitations to our study. First, the different types of ART applied, such as IVF-ET, IVF-FET, ICSI-ET and ICSI-FET, may also influence PTB risk. But we could not adjust for this important confounding factor due to lacking relevant information in the database. Second, the database did not provide GDM-relevant treatment or medication information, which might vary greatly from individual to individual. As maternal blood-sugar control can affect PTB, these may be important confounding factors in the current study. Third, potential for selection bias exists because the cohort was restricted to live births. Fourth, infection and inflammation during pregnancy can also be confounding factors that we could not account for. Additionally, we also cannot distinguish between spontaneous and medically induced PTB due to lack of relevant data.

## Conclusion

Our large cross-sectional study demonstrated ART pregnancies had a substantially increased risk for GDM compared with non-ART pregnancies, and ART mothers with GDM had the maximum risk of PTB, especially for mid-to-late PTB. As women receiving ART treatment were inherently at high risk of PTB by their age and comorbid conditions, this study highlights the importance of GDM prevention and controlling for the ART population.

## Data availability statement

The raw data supporting the conclusions of this article will be made available by the authors, without undue reservation.

## Author contributions

RG, JH, and CL conceived the idea. RG and KZ undertook data analysis. KZ, JZ, JH, and CL wrote draft of the manuscript. KZ, JZ, TL, SL, JL, and CL contributed to the interpretation of the results and critical revision of the manuscript for important intellectual content. All authors were involved in data analysis, drafting the article, or revising it critically for important intellectual content, and all authors approved the final version to be published.
