# Supplementary material for: Effects of gestational diabetes mellitus and assisted reproductive technology treatment on the risk of preterm singleton birth
Source: Front Nutr. 2022 Sep 14;9:977195. doi: 10.3389/fnut.2022.977195 (PMC9515569; doi:10.3389/fnut.2022.977195)
Supplement: Supplementary file 1 [file Data_Sheet_1.docx]

**
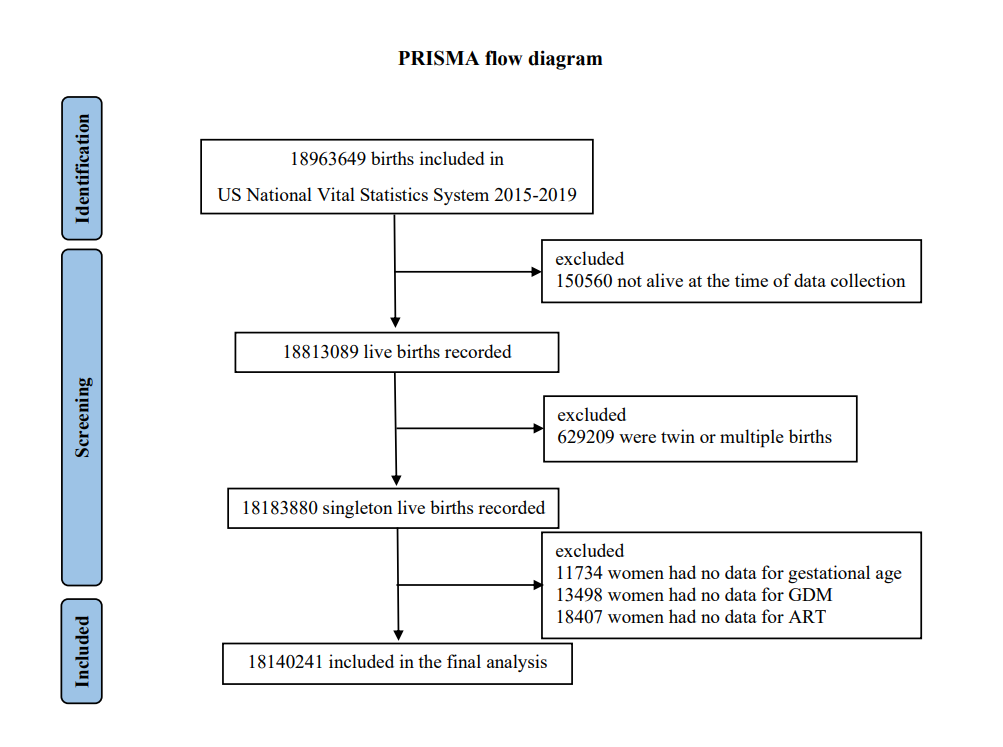
**

**Supplementary Figure 1. Flow chart of the screening process.** ART, assisted reproductive technology; GDM, gestational diabetes mellitus.

**Supplementary Table 1. Gestational diabetes mellitus among mothers who received assisted reproductive technology or not**

| **GDM** | **ART** | | **Total** | ***^2^*** | ***P*** |
| --- | --- | --- | --- | --- | --- |
|  | **Yes** | **No** |  |  |  |
| **Yes** | 15262 | 1110389 | 1125651 | 4618.21 | < 0.0001 |
| **No** | 130295 | 16884295 | 17014590 |  |  |
| **Total** | 145557 | 17994684 | 18140241 |  |  |

ART, assisted reproductive technology; GDM, gestational diabetes mellitus. Chi-square test was used.

**Supplementary Table 2. Characteristics of the study population, according to preterm birth**

| **Variables** | **None of ART or GDM** | | **ART only** | | **GDM only** | | **ART with GDM** | |
| --- | --- | --- | --- | --- | --- | --- | --- | --- |
|  | **FTB** | **PTB** | **FTB** | **PTB** | **FTB** | **PTB** | **FTB** | **PTB** |
| Overall | 15589132 | 1295163 | 116095 | 14200 | 985685 | 124704 | 13001 | 2261 |
| **Age, years, n (%)** | | | | | | | | |
| < 25 | 4115131(26.40) | 361015(27.87) | 1043(0.90) | 140(0.99) | 128868(13.07) | 16124(12.93) | 72(0.55) | 14(0.62) |
| 25-29 | 4634091(29.73) | 357808(27.63) | 10817(9.32) | 1401(9.87) | 249048(25.27) | 29505(23.66) | 810(6.23) | 136(6.02) |
| 30-34 | 4360738(27.97) | 335315(25.89) | 38480(33.15) | 4271(30.08) | 329339(33.41) | 39088(31.34) | 3567(27.44) | 581(25.70) |
| 35-39 | 2065514(13.25) | 191068(14.75) | 40971(35.29) | 4785(33.70) | 218155(22.13) | 29938(24.01) | 4985(38.34) | 773(34.19) |
| ≥ 40 | 413658(2.65) | 49957(3.86) | 24784(21.35) | 3603(25.37) | 60275(6.12) | 10049(8.06) | 3567(27.44) | 757(33.48) |
| **Race or ethnicity, n (%)** | | | | | | | | |
| Hispanic | 3714631(23.83) | 308540(23.82) | 9017(7.77) | 1369(9.64) | 256391(26.01) | 34748(27.86) | 1156(8.89) | 244(10.79) |
| Non-Hispanic Black | 8151844(52.29) | 591809(45.69) | 82670(71.21) | 9450(66.55) | 460219(46.69) | 51918(41.63) | 7556(58.12) | 1263(55.86) |
| Non-Hispanic White | 2181648(13.99) | 273797(21.14) | 5011(4.32) | 1102(7.76) | 107963(10.95) | 19938(15.99) | 595(4.58) | 154(6.81) |
| Other | 1541009(9.89) | 121017(9.34) | 19397(16.71) | 2279(16.05) | 161112(16.35) | 18100(14.51) | 3694(28.41) | 600(26.54) |
| **Education levels, n (%)** | | | | | | | | |
| Lower than high school | 2048270(13.14) | 210411(16.25) | 994(0.86) | 185(1.30) | 133940(13.59) | 18931(15.18) | 211(1.62) | 47(2.08) |
| High school | 3951832(25.35) | 374822(28.94) | 5937(5.11) | 890(6.27) | 232343(23.57) | 32171(25.80) | 862(6.63) | 172(7.61) |
| Higher than high school | 9396383(60.28) | 691287(53.37) | 105668(91.02) | 12679(89.29) | 606215(61.50) | 71925(57.68) | 11595(89.19) | 1982(87.66) |
| Missing | 192647(1.24) | 18643(1.44) | 3496(3.01) | 446(3.14) | 13187(1.34) | 1677(1.34) | 333(2.56) | 60(2.65) |
| **Marital status, n (%)** | | | | | | | | |
| Yes | 8629831(55.36) | 610235(47.12) | 96963(83.52) | 11791(83.04) | 599732(60.84) | 70535(56.56) | 10605(81.57) | 1829(80.89) |
| No | 5821224(37.34) | 604544(46.68) | 7769(6.69) | 1170(8.24) | 307493(31.20) | 45538(36.52) | 915(7.04) | 201(8.89) |
| Missing | 1138077(7.30) | 80384(6.21) | 11363(9.79) | 1239(8.73) | 78460(7.96) | 8631(6.92) | 1481(11.39) | 231(10.22) |
| Parity, n (%) | | | | | | | | |
| 1 | 6017483(38.60) | 504313(38.94) | 69723(60.06) | 9047(63.71) | 327751(33.25) | 42107(33.77) | 7970(61.30) | 1452(64.22) |
| 2 | 5038448(32.32) | 356499(27.53) | 31867(27.45) | 3346(23.56) | 314000(31.86) | 35002(28.07) | 3408(26.21) | 508(22.47) |
| 3 | 2630397(16.87) | 219291(16.93) | 8880(7.65) | 997(7.02) | 186050(18.88) | 23542(18.88) | 962(7.40) | 175(7.74) |
| ≥ 4 | 1855091(11.90) | 209787(16.20) | 5432(4.68) | 782(5.51) | 156170(15.84) | 23730(19.03) | 641(4.93) | 124(5.48) |
| Missing | 47713(0.31) | 5273(0.41) | 193(0.17) | 28(0.20) | 1714(0.17) | 323(0.26) | 20(0.15) | 2(0.09) |
| **Pre-pregnancy BMI, n (%)** | | | | | | | | |
| Normal weight | 6802829(43.64) | 503184(38.85) | 61269(52.77) | 6188(43.58) | 250506(25.41) | 25449(20.41) | 4528(34.83) | 630(27.86) |
| Obesity | 3862473(24.78) | 371535(28.69) | 20980(18.07) | 3573(25.16) | 442218(44.86) | 64026(51.34) | 4426(34.04) | 918(40.60) |
| Overweight | 4018914(25.78) | 314459(24.28) | 29097(25.06) | 3750(26.41) | 256518(26.02) | 29671(23.79) | 3649(28.07) | 636(28.13) |
| Underweight | 520832(3.34) | 54893(4.24) | 2861(2.46) | 305(2.15) | 15220(1.54) | 1787(1.43) | 214(1.65) | 30(1.33) |
| Missing | 384084(2.46) | 51092(3.94) | 1888(1.63) | 384(2.70) | 21223(2.15) | 3771(3.02) | 184(1.42) | 47(2.08) |
| **Pre-pregnancy hypertension, n (%)** | | | | | | | | |
| No | 15365191(98.56) | 1235683(95.41) | 113337(97.62) | 13318(93.79) | 250506(25.41) | 25449(20.41) | 12311(94.69) | 2048(90.58) |
| Yes | 223941(1.44) | 59480(4.59) | 2758(2.38) | 882(6.21) | 442218(44.86) | 64026(51.34%) | 690(5.31) | 213(9.42) |
| **Previous preterm birth, n (%)** | | | | | | | | |
| No | 9184705(58.92) | 655549(50.62) | 43357(37.35) | 4186(29.48) | 947271(96.10) | 113448(90.97) | 4581(35.24) | 622(27.51) |
| Yes | 386978(2.48) | 135333(10.45) | 3015(2.60) | 969(6.82) | 38414(3.90) | 11256(9.03) | 450(3.46) | 187(8.27) |
| Nulliparous | 6017449(38.60) | 504281(38.94) | 69723(60.06) | 9045(63.70) | 985685(0) | 124704(0) | 7970(61.30) | 1452(64.22) |
| **Smoking before pregnancy, n (%)** | | | | | | | | |
| No | 14152242(90.78) | 1123740(86.76) | 114629(98.74) | 13983(98.47) | 896485(90.95) | 111800(89.65) | 12770(98.22) | 2215(97.97) |
| Yes | 1352683(8.68) | 160814(12.42) | 1175(1.01) | 170(1.20) | 84801(8.60) | 12169(9.76) | 185(1.42) | 40(1.77) |
| Missing | 84207(0.54) | 10609(0.82) | 291(0.25) | 47(0.33) | 4399(0.45) | 735(0.59) | 46(0.35) | 6(0.27) |
| **Smoking during pregnancy, n (%)** | | | | | | | | |
| No | 14472963(92.84) | 1132023(87.40) | 115335(99.35) | 13728(96.68) | 919889(93.32) | 114088(91.49) | 12877(99.05) | 2218(98.10) |
| Yes | 507713(3.26) | 62370(4.82) | 232(0.20) | 41(0.29) | 29299(2.97) | 4419(3.54) | 48(0.37) | 9(0.40) |
| Missing | 608456(3.90) | 100770(7.78) | 528(0.45) | 431(3.04) | 36497(3.70) | 6197(4.97) | 76(0.58) | 34(1.50) |
| **Initiation of prenatal care, n (%)** | | | | | | | | |
| No prenatal care | 223775(1.44) | 64241(4.96) | 211(0.18) | 88(0.62) | 5027(0.51) | 1777(1.42) | 19(0.15) | 9(0.40) |
| 1th to 3th month | 11709332(75.11) | 923238(71.28) | 102438(88.24) | 12407(87.37) | 770888(78.21) | 96501(77.38) | 11413(87.79) | 1952(86.33) |
| 4th to 6th month | 2551345(16.37) | 199315(15.39) | 9961(8.58) | 1098(7.73) | 151505(15.37) | 18346(14.71) | 1162(8.94) | 212(9.38) |
| 7th to final month | 719770(4.62) | 47930(3.70) | 1711(1.47) | 123(0.87) | 40746(4.13) | 3675(2.95) | 214(1.65) | 26(1.15) |
| Missing | 384910(2.47) | 60439(4.67) | 1774(1.53) | 484(3.41) | 17519(1.78) | 4405(3.53) | 193(1.48) | 62(2.74) |
| **Gestational hypertension or preeclampsia & eclampsia, n (%)** | | | | | | | | |
| No | 14750828(94.62) | 1093395(84.42) | 106086(91.38) | 11037(77.73) | 880821(89.36) | 92360(74.06) | 11320(87.07) | 1642(72.62) |
| Yes | 838304(5.38) | 201768(15.58) | 10009(8.62) | 3163(22.27) | 104864(10.64) | 32344(25.94) | 1681(12.93) | 619(27.38) |
| **Infant sex, n (%)** | | | | | | | | |
| Female | 7936405(50.91) | 696026(53.74) | 58813(50.66) | 7709(54.29) | 507100(51.45) | 67791(54.36) | 6619(50.91) | 1204(53.25) |
| Male | 7652727(49.09) | 599137(46.26) | 57282(49.34) | 6491(45.71) | 478585(48.55) | 56913(45.64) | 6382(49.09) | 1057(46.75) |

ART, assisted reproductive technology; GDM, gestational diabetes mellitus; BMI, body mass index, FTB, full-term birth, PTB, preterm birth.

The percentage in the bracket was the composition ratio of each horizontal item.

Chi-square test showed that the inter-group *P* value of each variable was less than 0.05.

**Supplementary Table 3. Comparison of preterm birth between any two or more groups**

**among mothers who** **received assisted reproductive technology and/or developed gestational diabetes** **mellitus**

|  | | **Preterm birth** | | **Total** | ***^2^*** | ***P*** |
| --- | --- | --- | --- | --- | --- | --- |
|  |  | **No** | **Yes** |  |  |  |
| 1 | ART only | 116095(89.10%) | 14200(10.90%) | 130295 | 286.87 | < 0.0001 |
|  | GDM only | 985685(88.77%) | 124704(11.23%) | 1110389 |  |  |
|  | ART with GDM | 13001(85.19%) | 2261(14.81%) | 15262 |  |  |
| 4 | ART only | 116095(89.10%) | 14200(10.90%) | 130295 | 12.95 | < 0.0001 |
|  | GDM only | 985685(88.77%) | 124704(11.23%) | 130295 |  |  |
| 2 | ART only | 116095(89.10%) | 14200(10.90%) | 130295 | 208.90 | < 0.0001 |
|  | ART with GDM | 13001(85.19%) | 2261(14.81%) | 15262 |  |  |
| 3 | GDM | 985685(88.77%) | 124704(11.23%) | 1110389 | 193.24 | < 0.0001 |
|  | ART with GDM | 13001(85.19%) | 2261(14.81%) | 15262 |  |  |

ART, assisted reproductive technology; GDM, gestational diabetes mellitus. Chi-square test was used.
